# Smallholder farmers can achieve more sustainable wheat production through Consolidating Land for Uniform Practice

**DOI:** 10.3389/fpls.2025.1517683

**Published:** 2025-03-11

**Authors:** Taoyu Ren, Xue Yang, Wushuai Zhang, Wenhui Tang, Yajuan Li, Yinghao Tian, Jiawen Ren, Jun Yan, Xiaoxia Guo, Zhichao An, Hongyan Zhang

**Affiliations:** ^1^ State Key Laboratory of Nutrient Use and Management, College of Resources and Environmental Sciences, Key Laboratory of Plant-Soil Interactions, Ministry of Education, China Agricultural University, Beijing, China; ^2^ National Academy of Agriculture Green Development, China Agricultural University, Beijing, China; ^3^ College of Resources and Environment, Henan Agricultural University, Zhengzhou, China; ^4^ College of Resources and Environment, and Academy of Agricultural Science, Southwest University, Chongqing, China; ^5^ Quzhou Experimental Station, China Agricultural University, Handan, China

**Keywords:** smallholder agriculture, land fragmentation, land consolidation, multi-actor collaboration, wheat production

## Abstract

**Introduction:**

Land fragmentation of smallholder agriculture significantly constrains the adoption rate of optimal management practices and sustainable crop production.

**Methods:**

We developed and implemented an innovative management model known as Consolidating Land for Uniform Practice (CLUP), which aimed to foster multi-actor collaboration and facilitate the large-scale application of optimal practices without altering land ownership. CLUP was implemented in wheat fields in the North China Plain for three consecutive years.

**Results:**

Compared to conventional farmers’ practices (FP), the CLUP approach improved wheat yield by 14%, nitrogen recovery efficiency by 35%, net ecosystem economic benefit by 86%, and agricultural labor productivity by 53%. Additionally, greenhouse gas (GHG) emissions per hectare and per ton of grain were reduced by 18% and 32%, respectively. Although the wheat yield and environmental performance of CLUP were not as good as that of scientist-led optimal practices (SP), its agricultural labor productivity was 60% higher, and its economic cost was 10% lower than SP.

**Discussion:**

The CLUP model facilitates a transformative partnership by integrating the cutting-edge knowledge from universities, policy support from governments, and machinery services from enterprises, while emphasizing the participation of smallholder farmers. Overall, this study provides empirical evidence for optimizing agricultural practices and land management strategies, offering practical solutions for smallholder-dominated areas in the Global South.

## 1 Introduction

Smallholder farmers, despite cultivating only 24% of the world’s arable land, play a crucial role in food supply, hunger alleviation, and poverty reduction by producing 30-34% of the world’s foods (Ricciardi et al., 2018; Samberg et al., 2016). However, their agricultural practices have led to a series of significant resource and environmental burdens, such as soil acidification (Liu et al., 2023; Zhu et al., 2020), water eutrophication (Yu et al., 2019), global warming (Ming et al., 2024), and adverse impacts on human health (Hill et al., 2019). Additionally, it is concerning that smallholder farmers only achieve 54% of their potential yields, leaving a substantial yield gap, especially in developing countries (Yang et al., 2023). The adoption of sustainable agronomic practices, such as high-yield crop varieties and optimal water and fertilizer management, has been shown to reduce the yield gap while enhancing environmental and economic benefits (Wang et al., 2020; Yitayew et al., 2021). However, these practices are underutilized, with adoption rates among smallholder farmers below 40% (Zhao PF et al., 2016). Disseminating and adopting these practices is crucial for continuously feeding the growing global population while lowering the agricultural inputs.

Smallholder farmers face significant challenges in achieving sustainable agricultural transformation due to land fragmentation and inefficient technology adoption (Duan et al., 2021). Land fragmentation restricts the use of large-scale farm equipment, hindering the ability to implement advanced agricultural practices effectively. Additionally, the limited scale of their operations provides little economic incentive for smallholder farmers to optimize farming practices on their scattered fields. As a result, although China’s fragmented 13 million hectares of arable land contribute only 8% of the country’s crop production, they consume 15% of its nitrogen fertilizer (Deng et al., 2024). Furthermore, the adoption of advanced agricultural technologies, such as deep tillage and nitrogen fertilizer optimization, is often hindered by small farm sizes, lack of access to appropriate machinery, and limited knowledge and resources (Feng et al., 2020; Zhao et al., 2023). These barriers result in inefficient resource use, which limits productivity and sustainability (Ren et al., 2022; Zhao et al., 2022).

To address these issues, land consolidation and management transfer have been proposed as potential solutions to overcome the constraints posed by fragmented land. Large-scale farming has been demonstrated as an effective pathway to enhance resource efficiency and sustainability while maintaining productivity (Zhang et al., 2021). The Chinese government has actively encouraged land transfer through subsidies, concessional loans, and project funds for large-scale operators (Ju et al., 2016; Wang and Zhang, 2017). As a result, about one-third of small-scale farmlands have been transferred to large holders (Bartley, 2020). However, significant barriers remain, including smallholder farmers’ reluctance to transfer land due to concerns about losing access to farmland, limited alternative livelihood options, and inadequate compensation from the government (Gong et al., 2023; Wang et al., 2021). Thus, land consolidation without compromising the interests of smallholder farmers is a viable alternative. However, there is a lack of effective solutions to integrate land management, technology, policies, and products into a cohesive approach for sustainable agricultural transformation (Gorgan and Bavorova, 2022; Janus and Ertunç, 2022; Wu et al., 2023). In this context, the “one-stop agronomic service” approach offers a promising pathway. This model promotes multi-stakeholder collaboration to share resources, knowledge, and best practices, creating robust support systems tailored to the diverse needs of smallholder farmers. Through integrated services—such as training in modern practices, access to high-quality inputs, and facilitation of machinery and technology adoption—this approach can support the efficient and sustainable management of smallholder farms (Fieldsend et al., 2022; Xie et al., 2022; Garcia et al., 2021; Zhang et al., 2018). However, the effectiveness of such platforms in fragmented farming regions remains unclear, necessitating further exploration to fully realize their potential (Arnés et al., 2018; Zhao PF et al., 2016).

The North China Plain, dominated by smallholder agriculture, accounts for over 50% of China’s wheat production and sustains 60 million smallholder farmers, each with an average cultivating area of less than 0.25 ha (Cao et al., 2019; Wang et al., 2023; Zhao Y et al., 2016). However, fragmented land has led to a low adoption rate of advanced practices in wheat cultivation, resulting in excessive nutrient use and huge resource and environmental costs. Given the current wheat production situation in China and the practical needs of smallholder farmers, this study consolidates fragmented land and applies unified tailored agronomic practices without changing land property rights through multi-actor participation, aims to: (1) Integrate the Consolidating Land for Uniform Practice (CLUP) with multi-actor collaboration to build an innovative support model, making the advanced practices thoroughly being implemented on the ground; (2) Quantify the effectiveness of CLUP on yield, resource utilization, and greenhouse gas (GHG). In this study, we hypothesize that the CLUP model can integrate land management without altering land ownership, thereby effectively increasing yields and production efficiency while reducing environmental pollution. This study is expected to provide novel insights and practical evidence for regions dominated by smallholder agriculture worldwide, enabling smallholder farmers to achieve sustainable production without compromising their interests.

## 2 Methods

### 2.1 Site description

The study was conducted in Tianshui Village, Quzhou County in the North China Plain (**Supplementary Figure S1**). This region has a temperate semi-humid continental monsoon climate, with an annual average temperature of 13.1°C and a frost-free period of 201 days. The average annual precipitation is 542.8 mm, with a distinct wet season from late June to late September, during which 66.7% of the annual precipitation occurs. The warm climates support double cropping within a single year, with the predominant cropping system being winter wheat-summer maize rotation, where the wheat is generally sown in October and harvested in June of the following year. The soil texture comprises light loam, medium loam, sandy loam, clay and salt-affected. The basic properties of the surface layer soil (0–20 cm) are as follows: total nitrogen 940 mg kg^−1^ (from700 to 1060 mg kg^−1^), available phosphorus 23.0 mg kg^−1^ (Olsen-P, from 1.4 to 50.4 mg kg^−1^), and exchangeable potassium 132 mg kg^−1^ (Exc-K, from 67.1 to 190 mg kg^−1^) (**Supplementary Table S1**).

### 2.2 Consolidating land for uniform practice with multi-actor collaboration

**Figure 1:**
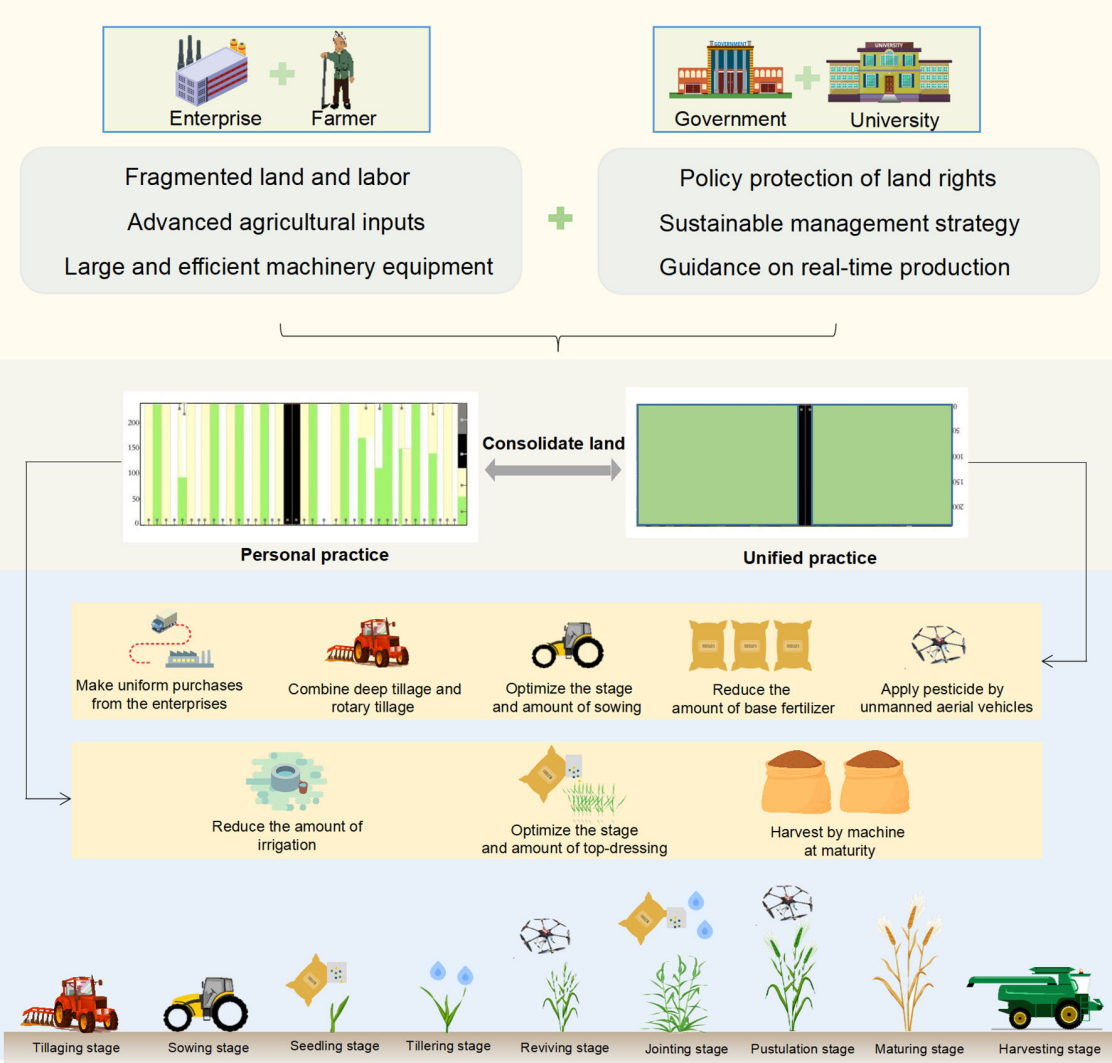
The schematic illustration of Consolidating Land for Uniform Practice (CLUP). The government, enterprises, scientists, and smallholder farmers provide policies, services, knowledge, organizations, land, and labor to carry out land consolidation and unified operations. By sharing resources, tailored practices package can be designed and applied on the farmlands to achieve higher yields with lower environmental burdens.

The CLUP was established by the Science and Technology Backyard (STB) – a platform established by China Agricultural University to foster information and knowledge exchange between the scientific institutions and farming communities. This project aims to collaborate smallholder farmers, local government, and agricultural enterprises, with the final goals of consolidating fragmented farmlands and implementing tailored practice packages (**Figure 1**). The CLUP implementation process involves five key steps:

Step 1: Determine the location. We selected a large-scale farmland consisting 23 adjacent plots from different smallholder farmers, covering a total area of 2.7 ha. We accordingly established CLUP on this farmland.

Step 2: Select the leading farmers. Farmers with extensive experience in cultivating farmlands and organizing activities were selected as leading farmers. They participated in CLUP activities either voluntarily or by invitation. Leading farmers played a crucial role in assisting CLUP staff to in adapting recommended management practices to local conditions and were responsible for overseeing field production.

Step 3: Consolidate farmlands. Government and leading farmers encouraged other smallholder farmers through meetings, broadcasts and home visits. Participation in CLUP was voluntary, with a commitment not to alter their land property rights.

Step 4: Develop tailored practice packages. Scientists and participating smallholder farmers got together to discuss and revise optimal practice proposed by scientists. The meetings, generally held by scientists, began with an introduction of the suggested practices, including their operating specifications, effects, and risks. This was followed by collecting feedbacks from smallholder farmers based on their practical experience and concerns. Finally, the tailored practice packages were determined through a bidirectional interaction and compromise between scientists and farmers.

Step 5: Implement tailored practice packages. The practice packages were jointly implemented by the multi-actors, including government, universities, enterprises, and smallholder farmers. Under government coordination, fragmented farmlands owned by smallholder farmers were consolidated into large-scale farmlands for demonstrating CLUP. There were two ways to implement tailored practice packages – unified operation and personal operation. For unified operation, leading farmers centrally managed activities such as land preparation, sowing, first fertilization, and plant protection. They coordinated the unified purchase of products, equipment, and services from enterprises. Consequently, economic costs were substantially reduced through bulk purchasing of seeds and fertilizers. Personal operation was generally applicable to labor-intensive activities, such as topdressing and irrigation, which were carried out by individual farmers. During crop production process, smallholder farmers received technical training and field guidance provided by STB staff. At the harvest time, all the CLUP activities were temporarily halted, allowing each individual to harvest independently and retain their own grains to avoid economic disputes that could affect the subsequent works. It is worth mentioning that throughout the entire production process, all participating farmers have the right to supervise the operations of CLUP.

To compare the effectiveness of CLUP, we set other two treatments – scientist practices (SP) and conventional farmer practices (FP) over the three consecutive years. SP adopted the best management practices (i.e., integrated soil-crop system management, ISSM) to achieve high-yield and high-efficiency in Quzhou Experimental Station (Chen et al., 2011). Plot size of SP was 1800 m^2^ with four replications. Simultaneously, we selected 20 adjacent farmlands (average 0.2 ha per household) with same soil conditions and similar sizes as those in CLUP for the FP treatment. The agricultural operations in FP treatment were carried out entirely according to farmers’ conventional practices. Both CLUP and FP conducted monitoring and sample collection in each of the 23 and 20 groups of participating farmers’ plots. Detailed management information is elaborated in **Table 1**.

**Table 1:** Comparations between farmers’ practice (FP), scientists’ practices (SP), and Consolidating Land for Uniform Practice (CLUP).

Practices	Characteristics	FP	SP	CLUP	Categories of CLUP
Agricultural purchase	Capital intensive	Independent purchase	Independent purchase	Unified purchase	Uniform
Land preparation	Mechanical intensive	Rotary tillage 2 times	Deep tillage 1 times; Rotary tillage 2 times; Depth of tillage:20-25 cm	Deep tillage 1 times; Rotary tillage 2 times; Depth of tillage:20-25 cm	Uniform
Sowing (Sowing date, sowing rate)	Mechanical intensive, Knowledge intensive	October 10^th^-15^th^, 268 kg ha^-1^	October 5^th^-8^th^, 168 kg ha^-1^	October 8^th^-9^th^, 189 kg ha^-1^	Uniform
Application of basal fertilizer (N rate)	Mechanical intensive, Knowledge intensive	126 kg ha^-1^	40 kg ha^-1^	89 kg ha^-1^	Uniform
Application of basal fertilizer (P_2_O_5_ rate)	Mechanical intensive, Knowledge intensive	128 kg ha^-1^	62 kg ha^-1^	95 kg ha^-1^	Uniform
Application of basal fertilizer (K_2_O rate)	Mechanical intensive, Knowledge intensive	79 kg ha^-1^	39 kg ha^-1^	49 kg ha^-1^	Uniform
Top-dressing (Top-dressing period and rate)	Labor intensive, Knowledge intensive	March 27^th^-April 12^th^, 124 kg ha^-1^	Early April, 122 kg ha^-1^	Early April, 107 kg ha^-1^	Personal
Irrigation (Irrigation frequency and amount)	Labor intensive, Knowledge intensive	3 times, 364 mm	3 times, 260 mm	3 times, 297 mm	Personal
Pesticide application (Implementation method)	Mechanical intensive, Knowledge intensive	Manual and agricultural unmanned aerial vehicle and application	Agricultural unmanned aerial vehicle^a^	Agricultural unmanned aerial vehicle	Uniform
Harvesting (Implementation method)	Mechanical intensive	Mechanical harvest at maturity	Mechanical harvest at maturity	Mechanical harvest at maturity	Personal

a The pesticide application was carried out on April 30 and May 16. The pesticides used were Triadimefon (with an active ingredient concentration of 10%, in wettable powder form) and Beta-cypermethrin (with an active ingredient concentration of 2.5%, in microemulsion form). The plant protection drone model used was ZFJN412.

Data in this table represents the three-year average, as the specific farming practices for each model had no significant variations over the three consecutive years. FP were all conducted personally. FP, Farmers’ practices; SP, Scientists’ practices; CLUP, Consolidating Land for Uniform Practice with multi-actor collaboration.

### 2.3 Data analysis

#### 2.3.1 N flow and N recovery efficiency

Poor nutrient management can result in high nitrogen losses (Dimkpa et al., 2020). In order to assess the impact of different models on the sustainability of nitrogen utilization, N flows in wheat production were calculated using the N input and output model. The N input included chemical N fertilizer (N_fert_), N from deposition (N_dep_), irrigation (N_irr_), seeds (N_seed_), and biological fixation (N_bio_).


(1) $$N_{input}=N_{fert}+N_{dep}+N_{irr}+N_{seed}+N_{bio}$$

In Equation 1, N_fert_ was calculated by multiplying the fertilizer application rate by the N concentration in the fertilizer. N_seed_ was calculated by multiplying the sowing amount by the concentration of N in the seeds. Referring to the results of Liu et al. (2016), N_dep_, N_irr_, and N_bio_ were set to 13 kg N ha^−1^, 13 kg N ha^−1^, and 15 kg ha^−1^, respectively.

The N output included N harvested in grain (N_up_), NH_3_ volatilization (N_NH3_), N leaching (N_leach_), N_2_O emissions (N_N2O_), and N accumulation in arable land (N_acc_). All straw was returned to the field in Quzhou, China.


(2) $$N_{output}=N_{up}+N_{NH3}+N_{leach}+N_{N2O}+N_{acc}$$

In Equation 2, N_up_, N_NH3_, N_leach_, and N_N2O_ rates were calculated by Cui et al. (2014) and Cui et al. (2018). The detailed calculation formulas are presented in **Supplementary Table S2**.

Nitrogen recovery efficiency (NRE) was used to further evaluate the productivity of cropping systems. The formula for calculating NRE is the ratio between N input and wheat grain harvest N (N_up_), as shown in Equation 3.


(3) $$N_{efficiency}=\frac{N_{up}}{N_{input}}\times 100\%$$

#### 2.3.2 Environmental impacts assessment

To assess the environmental impact of the CLUP model on wheat production, a systematic, region-specific life cycle assessment (LCA) was used (IPCC, 2006). In this study, the whole life cycle of agricultural material inputs (e.g., fertilizers, pesticides, diesel, and seeds) was evaluated from cradle to farmgate. The environmental impacts considered in this study included GHG emissions, soil acidification, and water eutrophication.

GHG emissions mainly origin from fuels, irrigation, fertilizers, and pesticides used in farming operations (Equation 4). The emission factors for agrochemical inputs during production and transportation were presented in **Supplementary Table S3**.


(4) $$\;GHG\;emissions=\sum_{i=1}^{n}EF_{i}\times Rate_{i}+N_{N2O}\times 44/28\times 265$$

where EF*_i_* is the emission factor for the *i* th agricultural input or chemical; Rate*_i_* refers to the amount of each input applied in wheat production. 44/28 is the coefficient for the conversion of N to N_2_O, and 265 is the coefficient of greenhouse equivalence of N_2_O compared to CO_2_ (IPCC, 2014).

For soil acidification potential (kg SO_2_-eq) and water eutrophication potential (kg PO_4_^-^eq), the calculation method is listed below:


(5) $$EI_{j}=\sum_{j=1}^{n}P_{ij}\times Rate_{j}$$

In Equation 5 where EI*_j_* is the *j*th impact category; Rate*_j_* represents the application rate of each input that was used in wheat production; P*_ij_* is the equivalent parameter of corresponding impact categories (**Supplementary Table S2**), which includes the production and application of nitrogen, phosphate, and potash fertilizers, as well as pesticides, diesel fuel, and electricity over the life cycle of the wheat system.

#### 2.3.3 Net ecosystem carbon budget

The net ecosystem carbon budget (NECB) is used to calculate the annual net ecosystem carbon balance, which indicates changes in soil organic carbon storage and shows whether a cropping system is a carbon sink or source (Mandal et al., 2021).


(6) $$NECB=\frac{C_{P}}{0.58}\left(GPP\right)-C_{G}-C_{E}+FRW$$

In Equation 6, C_P_ and C_E_ are the sums of all the carbon produced and consumed (the carbon emissions from wheat production), respectively. C_p_ includes the carbon content (Mg C per ha^−1^ yr^−1^) of grains (C_G_), straw (C_S_), root biomass (C_R_), and exudates (extra root, C_ER_), as per Equations 7–12 (Bolinder et al., 2007). GPP denotes gross primary productivity, and 0.58 is the conversion rate of GPP to C_P_ (Zhang et al., 2022a). FRW (field residue weight) was calculated by multiplying the carbon content and coefficient of field residue from the current crop season (1.22 in the study area, Wang et al., 2012).To evaluate the allocation of carbon within plant parts in a grain crop, we assumed that the C concentration of all plant parts was 0.45 g g^−1^ (Khorramdel et al., 2013).


(7) $$C_{P}=C_{G}+C_{S}+C_{R}+C_{ER}$$

(8) $$C_{E}=GHG\times 12/44$$

(9) $$C_{G}=Y_{P}\times 0.45$$

(10) $$C_{S}=Y_{P}\times \left(1-HI\right)/HI\times 0.45$$

(11) $$C_{R}=Y_{P}/\left(\frac{S}{R}\times HI\right)\times 0.45$$

(12) $$C_{ER}=C_{R}\times Y_{ER}$$

In Equations 7–12, Y*_P_* is the dry matter output of aboveground products (Mg ha^-1^ yr^-1^). HI is the harvest index, which is determined to be 0.417 based on measurement results. S/R is the shoot-root ratio, and Y_ER_, the extra root C (rhizo-deposit C) expressed as a factor relative to recoverable roots, is set to 7.4 and 0.65, respectively (Bolinder et al., 2007).

#### 2.3.4 Net ecosystem economic benefit

In order to clarify the economic viability and environmental sustainability of farmland of different models in wheat production, the net ecosystem economic benefit (NEEB) was employed by integrating the cost of agricultural activities and the damage costs of ecological and environmental destruction (Xia et al., 2016). The calculation of NEEB was based on the following formulas:


(13) $$NEEB=T_{benefit}-T_{cost}-Damage\;cost$$

(14) $$T_{cost}=\sum_{i=1}^{n}I_{i}\times P_{i}$$

(15) $$T_{benefit}=O_{grain}\times P_{grain}$$

(16) $$Damage\;cost=\sum_{i=1}^{n}ED_{i}\times P_{damage}$$

In Equations 13–16 where T*_cost_* includes the agricultural raw materials (seeds, fertilizers, pesticides, etc.) purchase cost and field management (electricity, diesel, labor, etc.) cost; I*_i_* is the input for wheat production, and P*_i_* is the unit price of the input (**Supplementary Table S4**). T*_benefit_* denotes the economic income per hectare of wheat. O_grain_ is the wheat yield (t), and P*_grain_* is the local commercial price (Chinese Yuan, CNY) of wheat in season. Damage cost refers to the economic loss caused by ecological damages (soil acidification, water eutrophication, hazardous to human health, climate warming), ED*_i_* refers to the *j*th impact category, and P*_damage_* represents the price coefficient that converts environmental damage into currency economic cost (**Supplementary Table S5**).

#### 2.3.5 Agricultural labor productivity analysis

The increase in the Agricultural labor productivity (ALP) is an important feature of the modernization and efficiency of agriculture, which was calculated as the ratio of yield to labor input (Zhang et al., 2020).


(17) $$ALP=\frac{O_{grain}}{L_{grain}}\times 100\%$$

In Equation 17 where O*_grain_* is the yield (t) of wheat and L*_grain_* is the total labor cost (h) at all stages of wheat production, including sowing, tillage, weeding, fertilizing, top-dressing, pesticide application, harvesting and straw mulching.

Origin (2018) and STAN (2.6.801) were used to draw graphs. Treatments were compared by single-factor analysis of variance using SPSS (IBM Statistics version 21) software. To identify significant treatment effects, multiple comparisons were performed with the least significant difference (LSD) test, and the significance level was set at the 0.05 probability level (*p*< 0.05).

## 3 Results

### 3.1 N flow and system productivity of different groups

**Figure 2:**
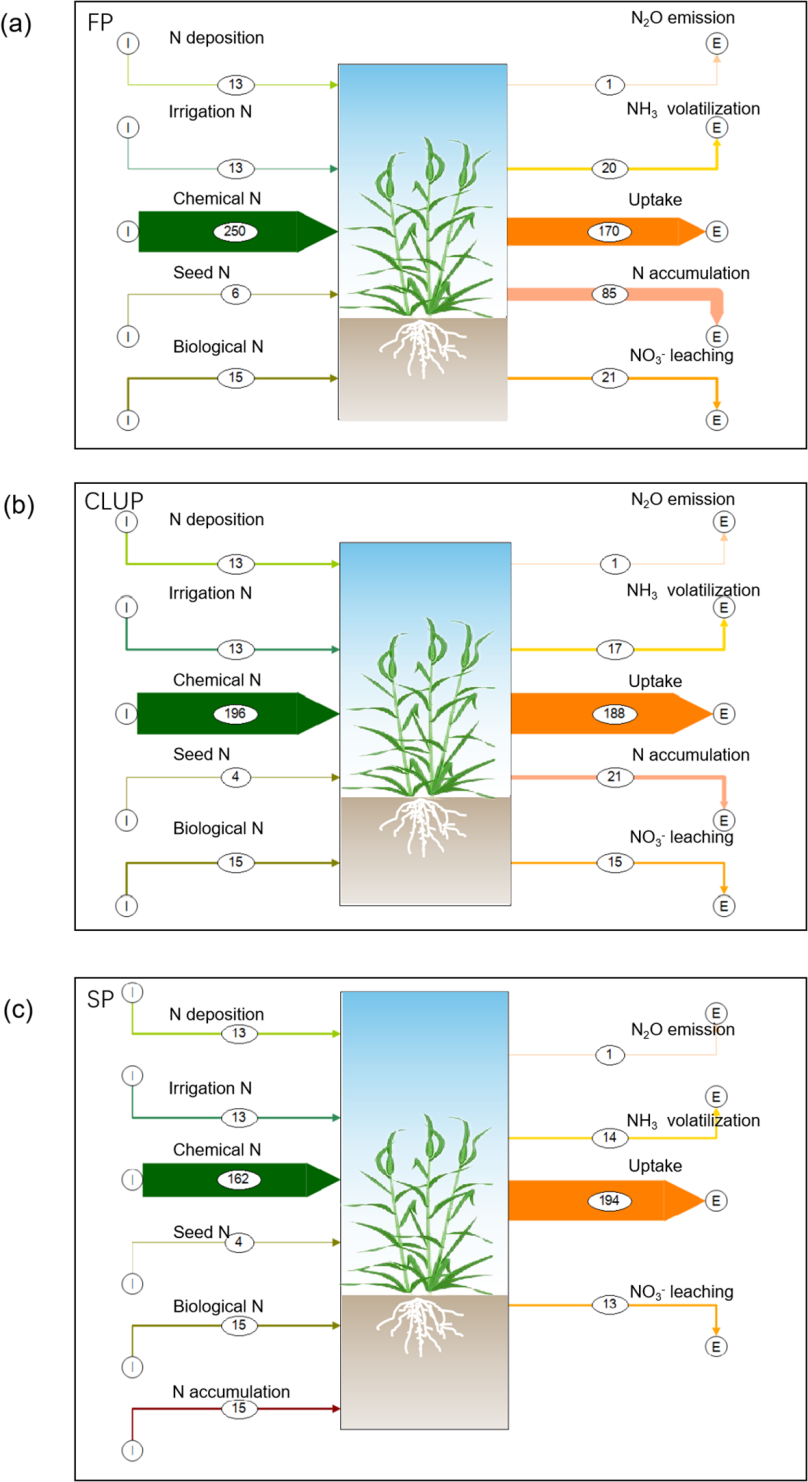
N flows (in kg) in wheat production systems. **(a)** Farmers’ practices (FP); **(b)** Consolidating Land for Uniform Practice (CLUP); **(c)** Scientists’ practices (SP).

Significant variations were observed in the N flow of wheat production among the three groups (**Figure 2**). The total N input in FP, which included deposition, irrigation, chemical fertilizer, seed and biological fixation, reached 298 kg ha^-1^, with 84% derived from chemical fertilizers. In comparison, N input in the CLUP and SP was reduced by 19% and 31% respectively, primarily due to notable reductions in fertilizer inputs (22% and 35%). Additionally, SP effectively utilized up to 15 kg ha^-1^ of residual nitrogen in the soil, thus mitigating the risk of nitrogen surplus. While wheat nitrogen uptake in CLUP (188 kg ha^-1^) was slightly lower than SP (194 kg ha^-1^), it significantly exceeded the FP by 11%. Notably, Nr loss (comprising N leaching, N_2_O emissions, NH_3_ volatilization, and N accumulation) was substantially lower in CLUP and SP (53 kg ha^-1^ and 12 kg ha^-1^, respectively) compared to FP (127 kg ha^-1^).

**Figure 3:**
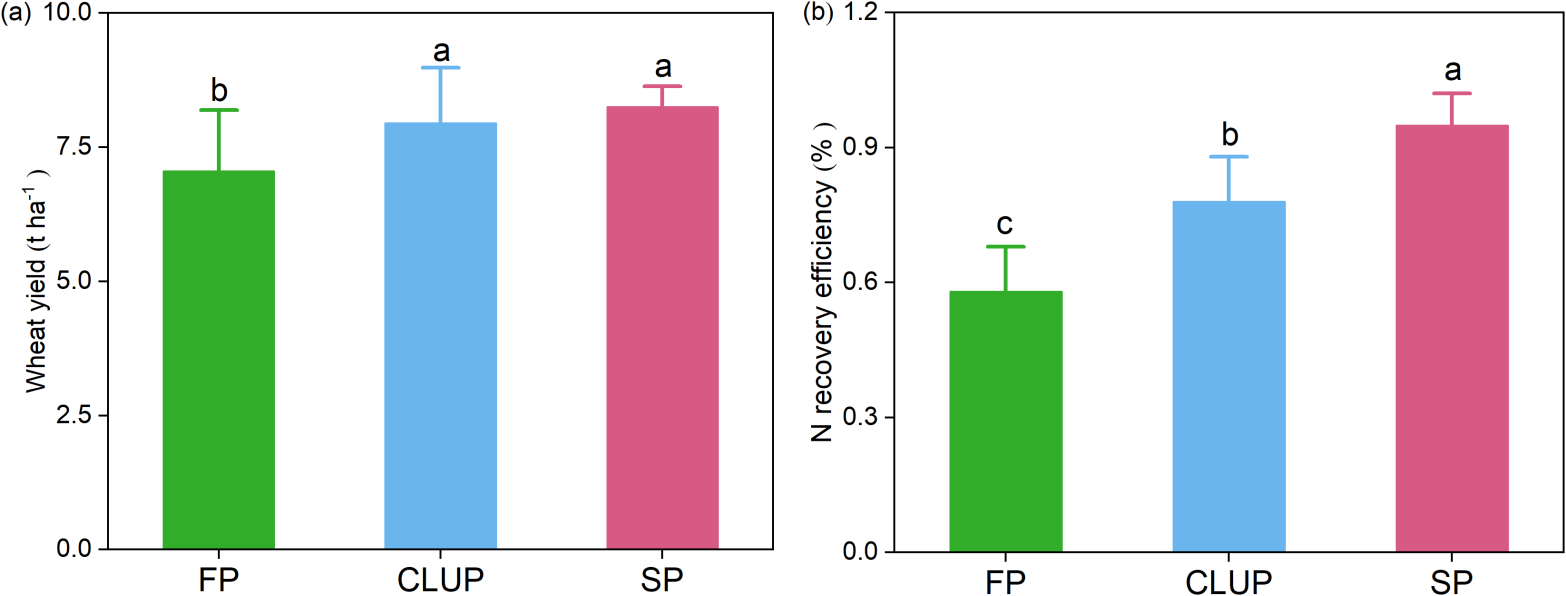
Wheat yield **(a)** and N recovery efficiency **(b)** under three management practices. FP, Farmers’ practices; CLUP, Consolidating Land for Uniform Practice; SP, Scientists’ practices. Different letters above the bars indicate significant differences among treatments at *p*< 0.05.

Higher grain yield and N recovery efficiency were observed in CLUP and SP (**Figure 3**). Specifically, grain yield in CLUP and SP were 8 t ha^-1^ and 8 t ha^-1^, marking increases of 13% and 17% respectively compared to FP. N recovery efficiency was 58% in FP, while CLUP and SP achieved 78% and 95%, respectively. However, there still exists a 4% and 18% gap in yield and N recovery efficiency between CLUP and SP.

### 3.2 GHG emissions and NECB in different groups

**Figure 4:**
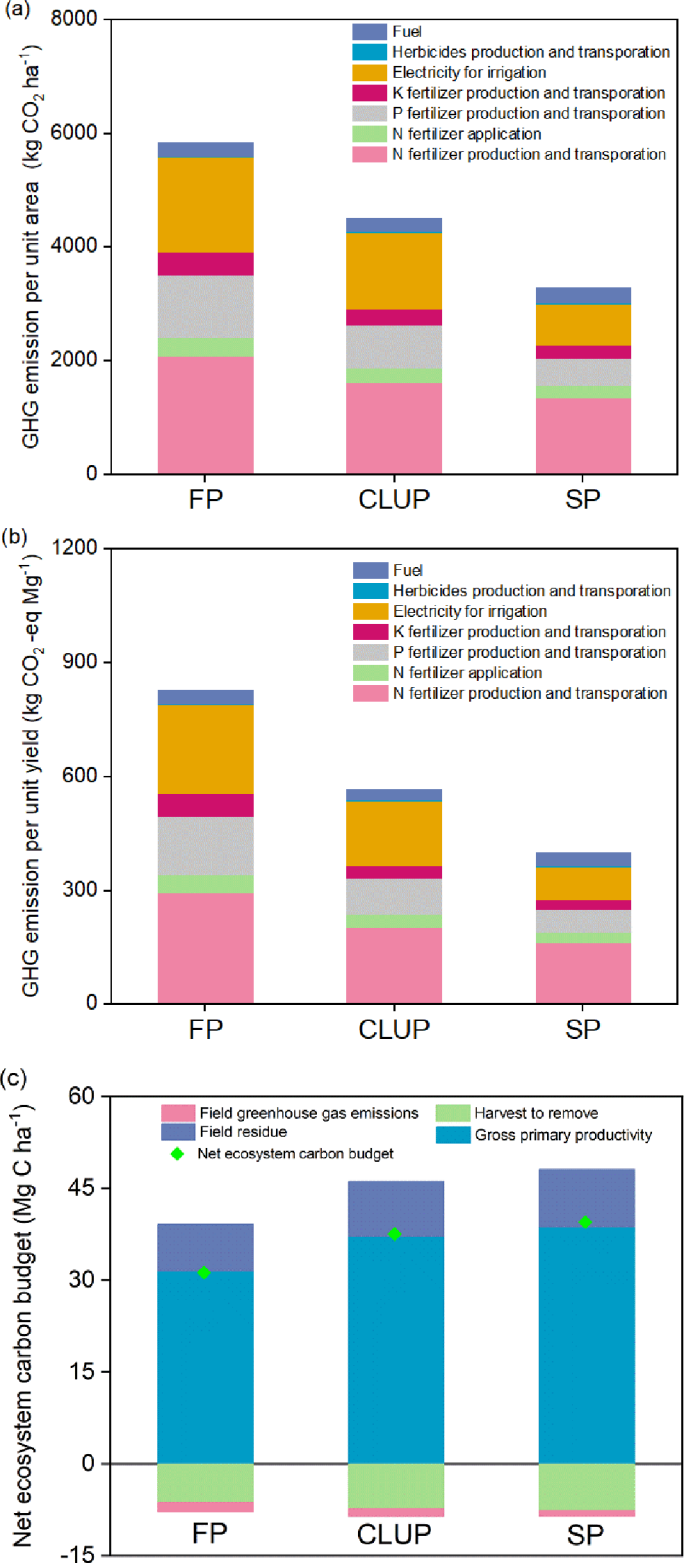
GHG emissions per unit area **(a)** and per unit yield **(b)**, and net ecosystem carbon budget **(c)** under three management practices. FP, Farmers’ practices; CLUP, Consolidating Land for Uniform Practice; SP, Scientists’ practices. Different letters above the bars indicate significant differences among treatments at *p*< 0.05.

The average GHG emissions per unit area of CLUP were 4497 CO_2_ ha^-1^, which fell between those of FP and SP (**Figure 4a**). The main sources of GHGs were nitrogen fertilizer production and electricity used for irrigation, contributing 34% to 41% and 22% to 34% of the total emissions across the three production models, respectively. Phosphate fertilizer application contributed an average of 16% of GHG emissions, followed by N fertilizer and K fertilizer, fuel, production and transportation of herbicide. Notably, GHG emissions from fuel consumption in CLUP were reduced by 19% compared to SP and by 14% compared to FP. FP had the highest GHG emissions per unit yield (827 kg CO_2_ eq-Mg ^-1^) among the three production models, with significant reductions of 32% and 52% for CLUP and SP, respectively (**Figure 4b**). The NECB ranged from 3 to 40 Mg C ha^-1^ across the three groups (**Figure 4c**), with CLUP showing a 20% increase compared to FP, mainly due to the significant increase in gross primary productivity.

### 3.3 Water eutrophication and soil acidification potential

**Figure 5:**
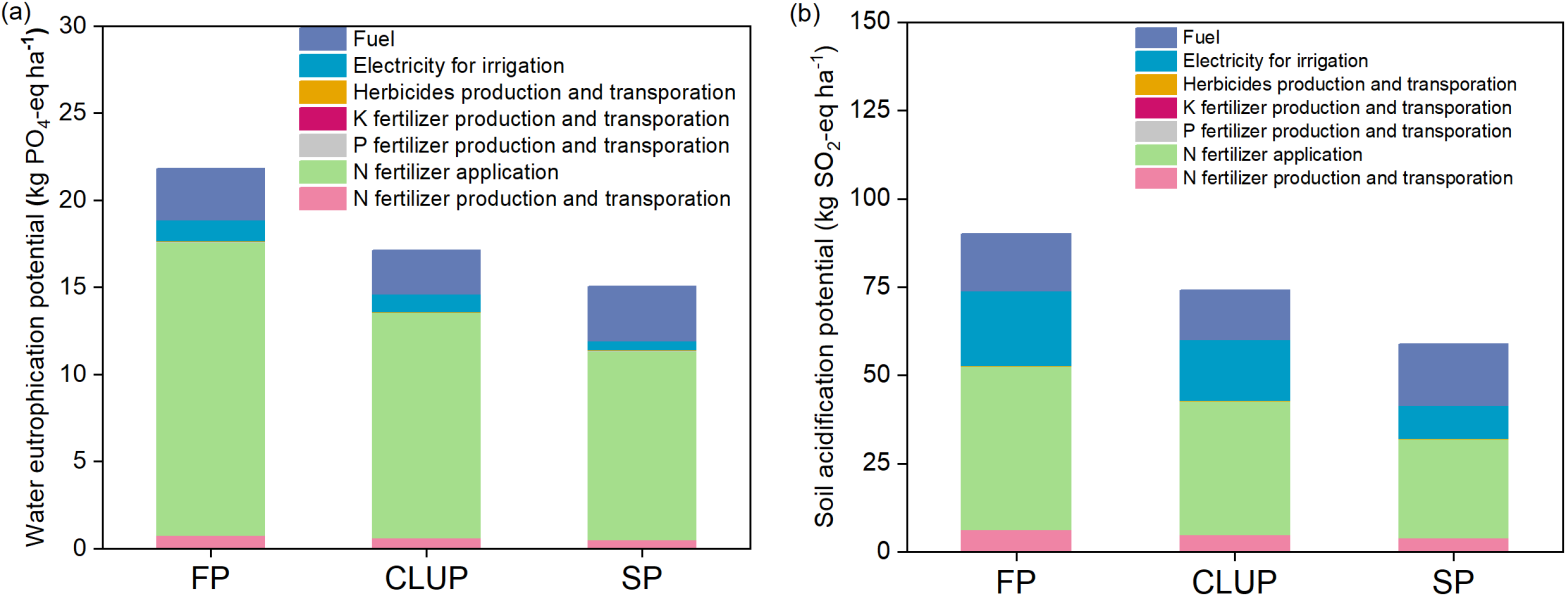
Water eutrophication potential **(a)** and soil acidification potential **(b)** under three management practices. FP, Farmers’ practices; CLUP, Consolidating Land for Uniform Practice; SP, Scientists’ practices. Different letters above the bars indicate significant differences among treatments at *p*< 0.05.

Similarly, the water eutrophication potential of CLUP was positioned between that of FP and SP (**Figure 5a**). CLUP’s water eutrophication potential (17 kg PO_4_-eq ha^-1^) decreased by 21% compared to FP (22 kg PO_4_-eq ha^-1^), but still showed a 15% difference compared to SP (15 kg PO_4_-eq ha^-1^). Nitrogen fertilizer application contributed 72% to 77% of PO_4_-eq across all three production models and was the main cause of water body eutrophication, followed by fuel consumption, which accounted for 13% to 21%.

The soil acidification potential followed a similar trend to water eutrophication (**Figure 5b**). CLUP’s soil acidification potential (74 kg SO_2_-eq ha^-1^) was 188% lower than that of FP. In three production models, Nitrogen fertilizer application emerged as the main contributor to soil acidification, accounting for 47% to 51%, followed by electricity used for irrigation. Notably, SP’s secondary source of soil acidification shifted to fuel, due to substantially reduced irrigation amounts, which constituted 39% of the total.

### 3.4 Economic and labor impact of different groups

**Figure 6:**
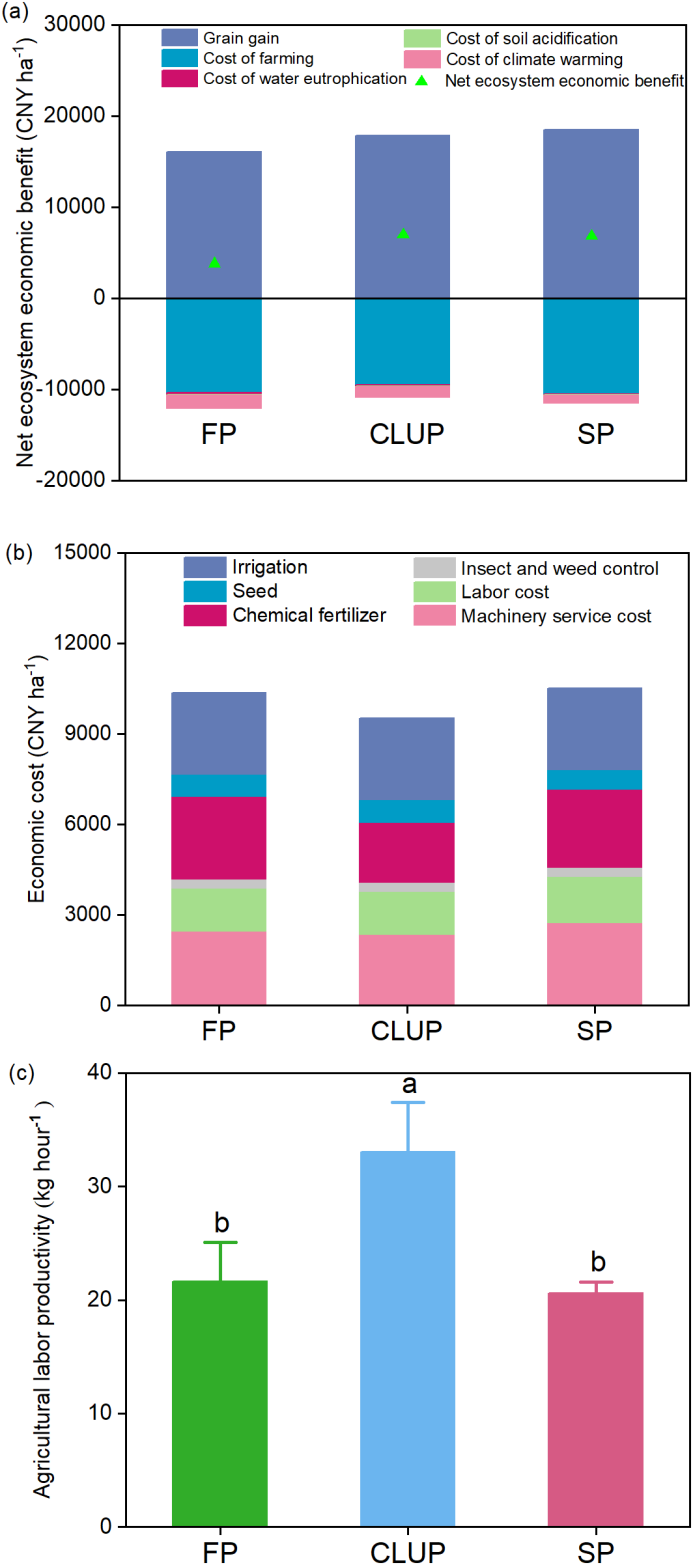
Net ecosystem economic budget **(a)**, cost **(b)**, and agricultural labor productivity **(c)** under three management practices. FP, Farmers’ practices; CLUP, Consolidating Land for Uniform Practice; SP, Scientists’ practices. Different letters above the bars indicate significant differences among treatments at *p*< 0.05.

CLUP achieved a commendable high net ecosystem economic budget of 6,986 CNY ha^-1^ with minimal cost and labor inputs, comparable to SP and FP (**Figure 6a**). This success was mainly due to high wheat yields and low environmental damage costs, including cumulative costs from greenhouse gas emissions, water eutrophication, and soil acidification.

Farming costs accounted for 84% to 89% of the total production cost across all three models. In the CLUP model, farming costs were 8% lower than FP and 9% lower than SP, mainly due to reduced fertilizer purchase prices (**Figure 6b**). Detailed cost expenditures are provided in **Supplementary Table S3**. Additionally, environmental damage costs in the CLUP model were 1,477 CNY ha^-1^, 28% lower than FP but 17% higher than SP. Remarkably, CLUP exhibited outstanding agricultural labor productivity, increasing by 53% compared to FP and 60% compared to SP (**Figure 6c**). This highlighted the efficiency gains achieved through CLUP adoption, streamlining operations, minimizing labor inputs, and maximizing productivity.

## 4 Discussions

### 4.1 Developing tailored practice packages based on smallholder farmers’ characteristics and land attributes

Smallholder farmers’ adoption of practices is influenced by various factors, involving challenges related to accessibility, usability, and understandability of technologies (Eastwood et al., 2020; George, 2014; Wens et al., 2021). Consequently, these challenges often result in low efficacy of technologies in smallholder field plots (Ayisi et al., 2022; Zhao PF et al., 2016). Engaging smallholder farmers in the process of innovating practices, combining scientific knowledge with smallholder farmers experience to develop tailored technologies, has proven effective in fostering farmers’ willingness to adopt new practices (Colnago et al., 2021; Fieldsend et al., 2022; Maryono et al., 2024). In this study, leading farmers, acting as farmer representatives, were invited from the outset to participate in working sessions aimed at developing practice packages. This approach provided them with a clear vision and understanding of the various management programs being developed (Wellens et al., 2013). Concurrently, scientists gained insights into the field realities that needed integration into the programs. The practice was iteratively refined through dialogue between scientists and farmers, striking a balance between their respective needs. For instance, the nitrogen fertilizer amount was adjusted from the recommended 162 kg N ha^-1^ to 196 kg N ha^-1^. This modification, although suboptimal, stayed below the FP average of 250 kg N ha^-1^ and addressed farmers’ concerns about potential yield penalties from reduced chemical nitrogen use (**Table 1**). Through this typical participatory research, smallholder farmers were able to access, use and understand the practice. Field application results showed that the revised practice system resulted in a 13% increase in grain yield, a 35% improvement in nitrogen recovery efficiency, and a 18% reduction in GHG emissions compared to FP. This evidence underscores that significant potential of the model to enhance the sustainability of crop production for predominantly smallholder farmers.

Implementing advanced agronomic practices on the fragmented lands is crucial for achieving sustainable production among smallholder farmers (Duan et al., 2021; Zhang et al., 2022b). Agricultural intensification has been identified as a potential solution (Maryono et al., 2024; Sánchez et al., 2018). Most studies have examined the models and effectiveness of large-scale operations through land transfer. For example, Zhang et al. (2022b) combined public-private partnerships with large-scale farming, a model that resulted in a 12% increase in yield and a 33% reduction in carbon footprint. Similarly, Zhang et al. (2022a) proposed a comprehensive straw management strategy for smallholders, which led to a 10% increase in yield. However, these solutions were difficult to replicate due to the low willingness of smallholder farmers to transfer land, particularly in developing countries (Wang et al., 2023; Xu et al., 2023). Therefore, innovating large-scale land management while safeguarding the land rights and interests of smallholder farmers is crucial to facilitate the adoption of advanced practices.

This study divided field operations into unified practices and personal practices, considering the characteristics of each practice and the specific needs of farmers. Unified practices involved overcoming land constraints and centralizing agronomic practices at scale across all farmer plots, coordinated by scientists and leading farmers. This approach aimed to ensure the application of mechanized ploughing, sowing, and drone spraying in fragmented plots, and alleviate economic costs associated with their application. Practices that did not require large-scale farm machinery, such as irrigation and top-dressing, were conducted by farmers themselves according to the recommended practice packages. During harvesting, farmers independently harvested their plots to protect their interests. This model facilitated large-scale operations without necessitating land transfer, thereby preserving land property rights. Overall, the model effectively integrates farmers’ needs, technological advancements, and land characteristics. It combines theoretical knowledge and operational practice packages, facilitating the integration of fragmented land ownership and the application of large-scale practice. As such, it serves as a convincing and successful example of achieving sustainable production despite the restrictions of land transfer.

### 4.2 Establishing transformative partnerships

Establishing transformative partnerships is crucial for successfully implementing advanced practices on a large scale across fragmented farmlands. This involves not only enhancing farmers’ willingness to adopt practices but also establishing a comprehensive support system. Such support system includes policy guarantees for land rights, access to advanced practices, and provision of large machinery and equipment (Adnan et al., 2019). However, these requirements often exceed the capacity and resources of individual smallholder farmers (George, 2014).

In the CLUP, key stakeholders underwent significant role transformations to enhance collaboration and ensure the project’s success. Traditionally, government-led initiatives have been characterized by top-down approaches, often leading to inefficiencies and failing to address the real needs of farmers (Zhang et al., 2018). However, in this study, the government transitioned from a dominant regulatory role to a supportive one, focusing on securing land tenure and introducing quality control measures, such as labeling on agricultural inputs to protect farmers from substandard products. Similarly, private enterprises shifted from supplying generic agricultural inputs to offering tailored products and services that better met farmers’ specific needs. This transformation not only promoted economic development within the private sector but also built trust between service providers and farmers. Moreover, scientists evolved from merely offering technical recommendations to becoming active collaborators, working closely with farmers to solve field-specific challenges. Moreover, farmers involved in CLUP took on supervisory and organizational roles within the project, mitigating the potential for profit-driven opportunism in the private sector (Yin et al., 2024). These changes in stakeholder roles created a more effective and mutually beneficial multi-actor partnership, driving both productivity and sustainability in smallholder agriculture.

Under the collaborative efforts of multi-actor partnership, advanced agricultural technologies have been implemented among smallholder farmers. Optimizing nitrogen fertilizer application played a key role in enhancing both crop yield and environmental sustainability. By adjusting the timing and amount of nitrogen applied, the nitrogen supply was better synchronized with the crop’s growth stages. This ensured that nutrients were available during critical periods, when crops could most efficiently utilize them, while minimizing losses through leaching and volatilization (Tian et al., 2024). In addition to fertilizer optimization, changes in tillage practices further contributed to both increased productivity and reduced environmental impact. The shift from traditional rotary tillage to a combination of deep tillage followed by rotary tillage enhanced soil structure by improving water infiltration and root growth. This not only allowed for more efficient nutrient uptake but also increased soil moisture retention, reducing the need for excessive irrigation (Feng et al., 2020). As a result, water use was optimized, leading to better resource management and a reduction in energy use for irrigation. The adoption of mechanized farming practices, such as large-scale sowing machinery and drone-based pesticide spraying, provided another layer of improvement. Mechanized sowing increased planting efficiency, allowing farmers to meet the recommended sowing window, even with limited labor availability. This helped align sowing with local climatic conditions, resulting in better crop establishment and higher yields. Additionally, drone-based pesticide spraying reduced labor inputs and ensured precise application, thereby minimizing chemical runoff and the overuse of pesticides, which further decreased environmental pollution (Arakawa and Kamio, 2023).

**Figure 7:**
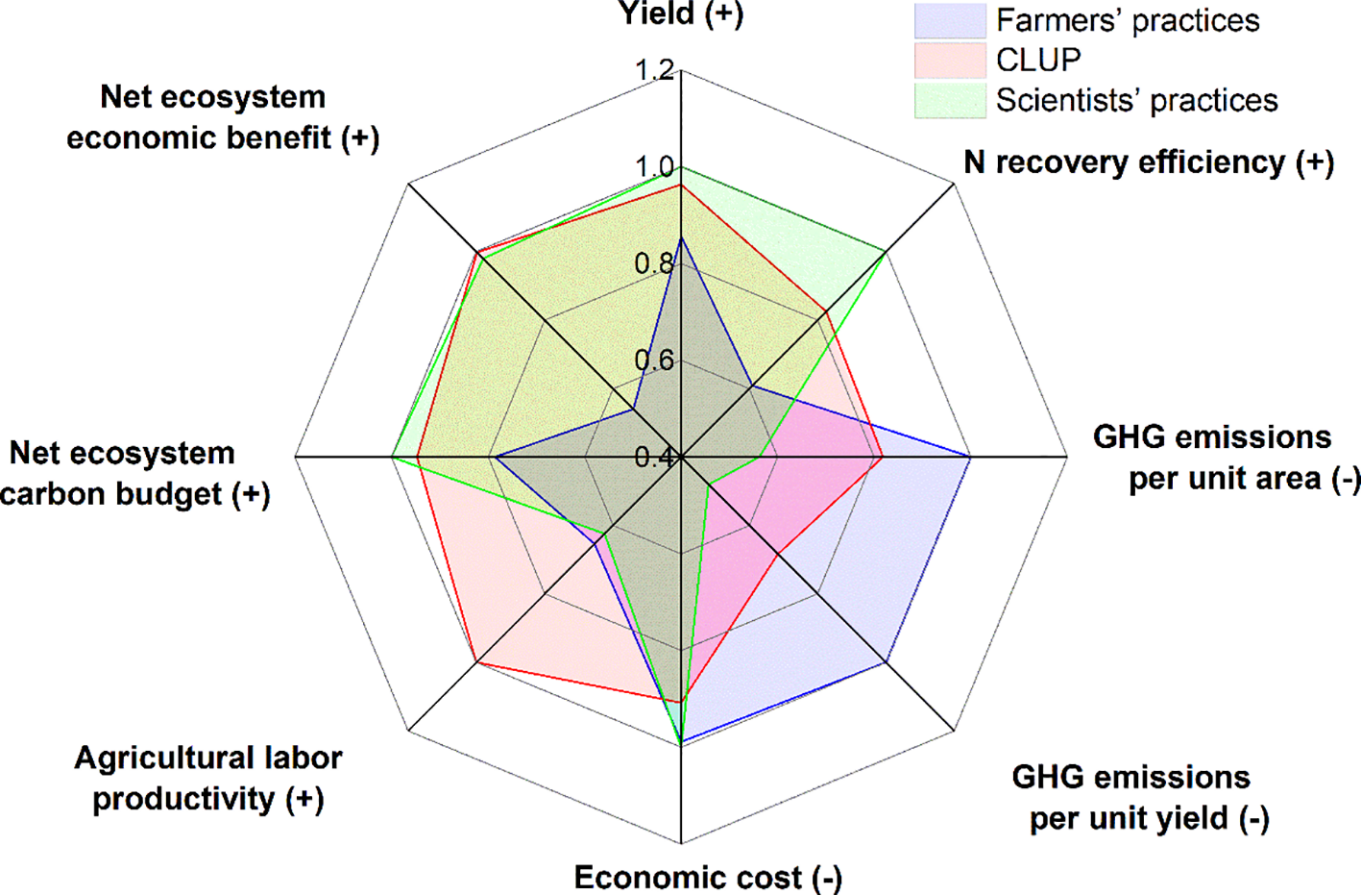
Multiple-objective comparisons of wheat production under three production models. “(+)” means that higher values indicate better performance. “(-)” means that lower values indicate better performance.

Ultimately, multi-objective crop production, grain yield, N recovery efficiency, net ecosystem economic benefit and agricultural labor productivity were improved by 14%, 35%, 86%, and 53%, respectively, and GHG emissions per unit area and per unit yield were reduced by 18% and 32%, respectively, highlighting the effectiveness of tailored agronomic practices facilitated by multi-actor participation (**Figure 7**). The transformative partnership forged through CLUP integrates cutting-edge crop nutrition knowledge and policy backing from the public sector with efficient agricultural machinery services provided by the private sector. This approach transcends mere collaboration, it represents a comprehensive strategy for sustainable intensification in crop production dominated by smallholder farmers, representing a significant stride towards agricultural sustainability.

### 4.3 Implementation and policy implications of CLUP

Globally, 500 million smallholder farmers contribute 28% of the food supply at a huge environmental cost (Bizikova et al., 2020; Liu, 2023). Enhancing their yields, incomes, and environmental performance is crucial for ensuring global food security and sustainable development (Yin et al., 2024; Zhang et al., 2016). Beyond China, other rapidly developing economies such as India, Brazil, and Indonesia are also facing similar conditions and challenges to transform smallholder agriculture towards sustainability (Mueller et al., 2013; Ricciardi et al., 2018). Multi-actor collaboration has gained increasing attention, such as Farmer Field Schools in Asia and Africa, the “LIAISON” project in Europe, and Soil Health Cards in India (Smyth et al., 2021; Fieldsend et al., 2022; Reddy, 2019). This study demonstrates a compelling example of multi-actor collaboration in achieving large-scale application of optimal management practices, thereby promoting sustainable intensification of agriculture. Consequently, CLUP has great potential for widespread implementation across various countries and regions.

Besides the traditional “top-down” strategy (e.g., policy incentives and financial support), a “bottom-up” approach is more crucial for implementing CLUP (Ensor and de Bruin, 2022). This approach relies on educating and raising awareness among farmers themselves. The STB organized training programs and workshops to disseminate information on best management practices (e.g., tillage and fertilization methods) and encourage farmers to participate in CLUP. Addressing actual needs and interests of farmers is essential for adopting innovative technologies. Guiding farmers to act as co-designers of technological innovations, rather than passive recipients, is vital for the rapid adoption of advanced technologies (Cui et al., 2018; Zhang et al., 2016; Mariano et al., 2012). Moreover, leading farmers play a vital role in disseminating optimal practices, serving as a conduit for advanced technologies, and connecting farmers with research institutions.

### 4.4 Limitations and further perspectives

This study has two major limitations. First, the scale of its application is relatively small, because it was designed primarily as an exploratory experiment to demonstrate the feasibility and effectiveness of CLUP. In future studies, we will apply the CLUP model in other smallholder-dominated areas to verify its replicability and provide empirical evidence for subsequent upscaling. Additionally, this study focused primarily on the environmental impacts of nitrogen fertilizer application. Future research should encompass a broader and more comprehensive range of environmental impacts to provide a more accurate assessment of the CLUP model’s effectiveness.

Thirdly, despite our efforts to develop tailored agronomic practices and build a transformative partnership for the application of tailored practices, gaps still existed between CLUP and SP in terms of multi-objective coordination. It is critical to recognize that this process requires multiple rounds of improvement to continuously bridge the gap between CLUP and SP. Additionally, more innovations (e.g., collaboration methods, consolidation methods of fragmented farmlands, and involvement of more stakeholders) are needed to develop various flexible solutions for other regions worldwide with diverse endowment and situations.

## 5 Conclusions

In this study, we integrated the resources from multi-actors through a transformative partnership to foster the adoption of optimal management practices by smallholder farmers. We firstly established a system-based tailored large-scale practice application and land consolidation model, called Consolidating Land for Uniform Practice (CLUP), and applied it in Quzhou County in the North China Plain. The results demonstrated that CLUP significantly increased grain yield, N recovery efficiency, net ecosystem economic benefit and agricultural labor productivity of the wheat systems by 14%, 35%, 86%, and 53%, respectively, while reducing GHG emissions per unit area and per unit yield by 18% and 32%. Although CLUP caused greater environmental impacts than SP, it better met the actual needs and acceptance of smallholder farmers for future intensification and sustainable development. Therefore, it is viable that through the participation of multiple actors, suboptimal smallholder management can be transformed into more sustainable management. This work also provides empirical evidence for policymakers, university researchers, and private-sector leaders to formulate policies and decisions to improve sustainability in areas dominated by smallholder farmers.

## Data Availability

The raw data supporting the conclusions of this article will be made available by the authors, without undue reservation.
