# Supplementary material for: Smallholder farmers can achieve more sustainable wheat production through Consolidating Land for Uniform Practice
Source: Front Plant Sci. 2025 Mar 11;16:1517683. doi: 10.3389/fpls.2025.1517683 (PMC11933068; doi:10.3389/fpls.2025.1517683)
Supplement: Supplementary file 2 [file DataSheet1.docx]

Supplementary Information for

**Smallholder farmers can achieve more sustainable wheat production through Consolidating Land for Uniform Practice**

Taoyu Ren ^a,b^, Xue Yang ^c^, Wushuai Zhang ^d^, Wenhui Tang ^a,b^ , Yajuan Li ^a,b^, Yinghao Tian ^e^, Jiawen Ren ^a,b^, Jun Yan ^a,b^, Xiaoxia Guo ^a,b,*^, Zhichao An ^a,b,*^, Hongyan Zhang ^a,b^

^a^ State Key Laboratory of Nutrient Use and Management, College of Resources and Environmental Sciences, Key Laboratory of Plant-Soil Interactions, Ministry of Education, China Agricultural University, Beijing 100193, China

^b^ National Academy of Agriculture Green Development, China Agricultural University, Beijing 100193, China

^c^ College of Resources and Environment, Henan Agricultural University, Zhengzhou 450002, China

^d^ College of Resources and Environment, and Academy of Agricultural Science, Southwest University, Chongqing 400715, China

^e^ Quzhou Experimental Station, China Agricultural University, Handan 057250, China

^*^ Corresponding authors at: 2 Yuanmingyuan Xilu, Beijing 100193, China

E-mail addresses: guoxiaoxia@cau.edu.cn (X. Guo), [anzc@cau.edu.cn](mailto:anzc@cau.edu.cn) (Z. An)


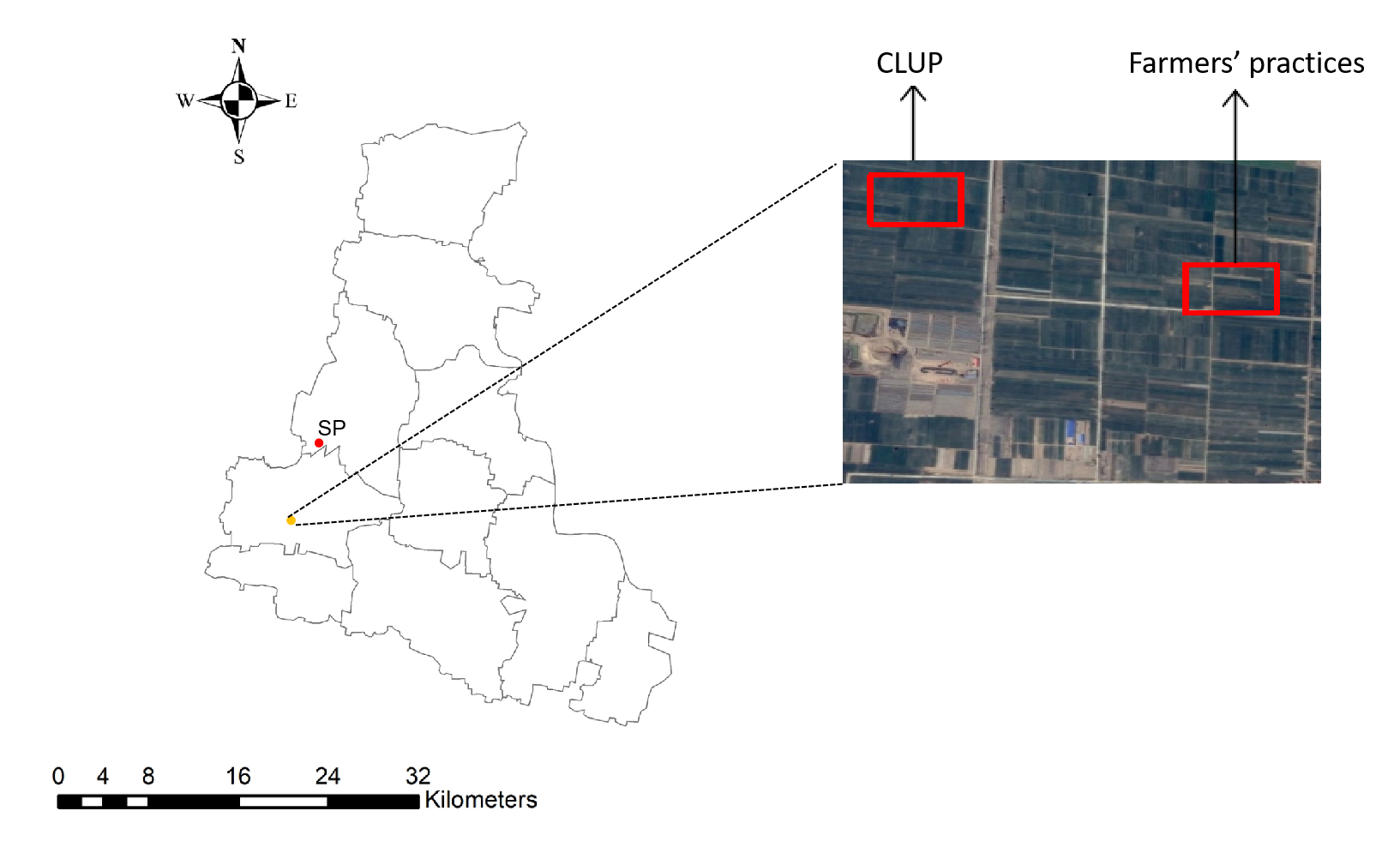


Fig. S1 Distribution Map of the Experimental Area in Quzhou County.

Table S1 Climatic Conditions and Soil Physicochemical Properties of the Experimental Area

| average temperature | frost-free period | average annual precipitation | total nitrogen | available phosphorus | exchangeable potassium |
| --- | --- | --- | --- | --- | --- |
| 13.1 °C | 201 days | 542.8 mm | 940 mg kg^−1^ | 23.0 mg kg^−1^ | 132 mg kg^−1^ |

Table S2 Calculation formula of N flow section parameters

| parameters | Calculation formula | References |
| --- | --- | --- |
| N_up_ | $N_{up}=-14+41\times Y^{0.77}$^a^ | Cui et al., 2014 |
| N_NH3_ | $N_{NH3}=3.21+0.068\times N_{fert}$ | Cui et al., 2018 |
| N_leach_ | $N_{leach}={4.93e}^{0.0057Nfert}$ | Cui et al., 2018 |
| N_N2O_ | $N_{N2O}={0.26e}^{0.0045Nfert}$ | Cui et al., 2018 |

^a^Y is grain yield

Table S3 GHG emission factors of agricultural chemicals in production and transportation stage.

| Items | Unit | GHG emissions | Water eutrophication | Soil acidification | References |
| --- | --- | --- | --- | --- | --- |
|  |  | (kg CO_2_ eq unit^−1^) | (kg PO_4_ eq unit^−1^) | (kg SO_2_ eq unit^−1^) |  |
| N fertilizer production and transportation | kg N | 8.30 | 0.00303 | 0.0252 | Di et al., 2005; Yuan et al., 2006; Esengun et al., 2007; Zhang et al., 2013; Yue, 2013 |
| P fertilizer production and transportation | kg P_2_O_5_ | 7.90 | 0.00008 | 0.0006 | Di et al., 2005; Yuan et al., 2006; Esengun et al., 2007; Zhang et al., 2013; Yue, 2013 |
| K fertilizer production and transportation | kg K_2_O | 5.50 | 0.00006 | 0.00048 | Di et al., 2005; Yuan et al., 2006; Esengun et al., 2007; Zhang et al., 2013; Yue, 2013 |
| Pesticide production and transportation | kg | 19.12 | 0.00194 | 0.0105 | Demircan et al., 2006; Cui et al., 2013; Williams, Audsley, and Sandar., 2006 |
| Diesel | L | 3.75 | 0.0119 | 0.0658 | IPCC, 2006; Yuan et al., 2006; Erdal et al., 2007; Cui et al., 2013 |
| Electricity for irrigation | kWh | 1.14 | 0.00084 | 0.0145 | Zhang et al., 2013; Yue., 2013; Asgharipour et al., 2012 |

Table S4 The detaileds cost (CNY)in the three different study groups. FP, Farmers' practices; CLUP, Consolidating land for Uniform with multi-actor collaboration; SP, Scientists' practices

|  | CLUP | FP | SP | Categories |
| --- | --- | --- | --- | --- |
| Crushing corn stalks | 675 | 750 | 750 | Machinery service cost |
| Basal Fertilizer | 1545 | 2175 | 650 | Chemical fertilizer |
| Land preparation | 225 | 300 | 400 | Machinery service cost |
| Seed | 750 | 750 | 650 | Seed |
| Machine Operator Supervisor | 225 | 225 | 225 | Labor cost |
| Sowing | 300 | 375 | 450 | Machinery service cost |
| Irrigation labor | 1125 | 1125 | 1125 | Labor cost |
| Irrigation electricity | 2700 | 2700 | 2700 | Irrigation |
| Herbicides | 75 | 75 | 75 | Insect and weed control |
| Weed control operating costs | 45 | 75 | 150 | Machinery service cost |
| Pest control agents | 225 | 225 | 225 | Insect and weed control |
| Pest control operating costs | 135 | 225 | 250 | Machinery service cost |
| Top Dressing | 450 | 550 | 1950 | Chemical fertilizer |
| Fertilizer labor | 75 | 75 | 175 | Labor cost |
| Harvesting | 675 | 750 | 750 | Machinery service cost |
| Break the ridge of the field | 300 | 0 | 0 | Machinery service cost |
| Total cost | 9525 | 10375 | 10525 |  |

Table S5 The damage costs (CNY) of various Nr species and GHG emissions to the ecosystems.human health and climate warming in China.

| Items | Unit | Ecosystems | Human health | Climate warming | References |
| --- | --- | --- | --- | --- | --- |
| NH_3_ volatilization | kg N | 13.2 | 24.3 |  | Xia et al.,2016;Gu et al., 2012;Van et al., 2013 |
| NO_x_ emission | kg N | 8.7 | 20.9 |  | Xia et al.,2016;Gu et al., 2012;Van et al., 2013 |
| N_2_O emission | kg N |  | 2.1 | 81.6 | Xia et al.,2016;Gu et al., 2012;Van et al., 2013 |
| N leaching and runoff | kg N | 7.9 | 1.4 |  | Xia et al.,2016;Gu et al., 2012;Van et al., 2013 |
| GHG emission | t CO_2_ -eq |  |  | 174.3 | Xia et al.,2016;Gu et al., 2012;Van et al., 2013 |

The damage costs to the ecosystems were mainly in relation to soil acidification and water eutrophication caused by NH_3_ volatilization and NOX emissions, and water eutrophication caused by N leaching and runoff.

Table S6a ANOVA and Post-Hoc Tukey HSD Results for Group Comparisons in yield.

| Comparison | Test Type | Test Statistic | Degrees  of Freedom | P-Value | Significance |
| --- | --- | --- | --- | --- | --- |
| ANOVA | One-Way ANOVA | F = 9.969 | df = 2113 | 0.000103 | *** |
| FP vs CLUP | Tukey HSD (Post-Hoc) | Difference = -0.884 | - | 0.0000974 | *** |
| SP vs CLUP | Tukey HSD (Post-Hoc) | Difference = 0.303 | - | 0.8839 | NS |
| SP vs FP | Tukey HSD (Post-Hoc) | Difference = 1.186 | - | 0.159 | NS |

Table S6b ANOVA and Post-Hoc Tukey HSD Results for Group Comparisons in N recovery efficiency.

| Comparison | Test Type | Test Statistic | Degrees of Freedom | P-Value | Significance |
| --- | --- | --- | --- | --- | --- |
| ANOVA | One-Way ANOVA | F = 70.67 | df = 2113 | < 2e-16 | *** |
| FP vs CLUP | Tukey HSD (Post-Hoc) | Difference = -0.205 | - | 0.0000000 | *** |
| SP vs CLUP | Tukey HSD (Post-Hoc) | Difference = 0.166 | - | 0.0141901 | * |
| SP vs FP | Tukey HSD (Post-Hoc) | Difference = 0.371 | - | 0.0000000 | *** |

Table S6c ANOVA and Post-Hoc Tukey HSD Results for Group Comparisons in GHG emissions.

| Comparison | Test Type | Test Statistic | Degrees of Freedom | P-Value | Significance |
| --- | --- | --- | --- | --- | --- |
| ANOVA | One-Way ANOVA | F = 541.9 | df = 2,113 | < 2e-16 | *** |
| FP vs CLUP | Tukey HSD (Post-Hoc) | Difference = 1346.36 | - | 0 | *** |
| SP vs CLUP | Tukey HSD (Post-Hoc) | Difference = -1211.77 | - | 0 | *** |
| SP vs FP | Tukey HSD (Post-Hoc) | Difference = -2558.13 | - | 0 | *** |

Table S6d ANOVA and Post-Hoc Tukey HSD Results for Group Comparisons in NECB.

| Comparison | Test Type | Test Statistic | Degrees of Freedom | P-Value | Significance |
| --- | --- | --- | --- | --- | --- |
| ANOVA | One-Way ANOVA | F = 15.4 | df = 2, 113 | 1.22e-06 | *** |
| FP vs CLUP | Tukey HSD (Post-Hoc) | Difference = -6294.17 | - | 0.0000012 | *** |
| SP vs CLUP | Tukey HSD (Post-Hoc) | Difference = 1941.64 | - | 0.8559298 | NS |
| SP vs FP | Tukey HSD (Post-Hoc) | Difference = 8235.81 | - | 0.0682113 | NS |

Table S6e ANOVA and Post-Hoc Tukey HSD Results for Group Comparisons in NEEB.

| Comparison | Test Type | Test Statistic | Degrees of Freedom | P-Value | Significance |
| --- | --- | --- | --- | --- | --- |
| ANOVA | One-Way ANOVA | F = 14.68 | df = 2, 113 | 2.16e-06 | *** |
| FP vs CLUP | Tukey HSD (Post-Hoc) | Difference = -2952.53 | - | 0.0000013 | *** |
| SP vs CLUP | Tukey HSD (Post-Hoc) | Difference = -79.79 | - | 0.9988144 | NS |
| SP vs FP | Tukey HSD (Post-Hoc) | Difference = 2872.73 | - | 0.2244527 | NS |

Table S6f ANOVA and Post-Hoc Tukey HSD Results for Group Comparisons in ALP.

| Comparison | Test Type | Test Statistic | Degrees of Freedom | P-Value | Significance |
| --- | --- | --- | --- | --- | --- |
| ANOVA | One-Way ANOVA | F = 119.8 | df = 2, 113 | <2e-16 | *** |
| FP vs CLUP | Tukey HSD (Post-Hoc) | Difference = -11.4362 | - | 0.0000000 | *** |
| SP vs CLUP | Tukey HSD (Post-Hoc) | Difference = -12.4127 | - | 0.0000020 | *** |
| SP vs FP | Tukey HSD (Post-Hoc) | Difference = -0.9765 | - | 0.9107434 | NS |

**References**

Asgharipour, M.R., Mondani, F., Riahinia, S., 2012. Energy use efficiency and economic analysis of sugar beet production system in Iran: A case study in Khorasan Razavi province. Energy 44, 1078-1084. <http://doi.org/10.1016/j.energy.2012.04.023.>

Cui, Z.L., Yue, S.C., Wang, G.L., et al, 2013. In-season root-zone N management for mitigating greenhouse gas emission and reactive N losses in intensive wheat production. Environ. Sci. Technol. 47, 6015-6022. <http://doi.org/10.1021/es4003026>.

Cui, Z.L., Wang, G., Yue, S., et al., 2014. Closing the N-Use Efficiency Gap to Achieve Food and Environmental Security. Environ. Sci. Technol. 48, 5780–5787. https://doi.org/10.1021/es5007127

Cui, Z.L., Zhang, H., Chen, X.P., et al., 2018. Pursuing sustainable productivity with millions of smallholder farmers. Nature. 555, 363–366. https://doi.org/10.1038/nature25785

Demircan, V., Ekinci, K., Keener, H.M., Akbolat, D., Ekinci, C., 2006. Energy and economic analysis of sweet cherry production in Turkey: A case study from Isparta province. Energ. Convers. Manage. 47, 1761-1769. <http://doi.org/10.1016/j.enconman.2005.10.003.>

Di, X.H., Nie, Z.R., Zuo, T.Y., 2005. Life cycle emission inventories for the fuels consumed by thermal power in China. China Environmental Science. 25(5), 632–635. <https://doi.org/10.3321/j.issn:1000-6923.2005.05.029>. (In Chinese).

Esengun, K., Gündüz, O., Erdal, G., 2007. Input–output energy analysis in dry apricot production of Turkey. Energ. Convers. Manage. 48, 592-598. <http://doi.org/10.1016/j.enconman.2006.06.006.>

Erdal, G., Esengün, K., Erdal, H., Gündüz, O., 2007. Energy use and economical analysis of sugar beet production in Tokat province of Turkey. Energy 32, 35-41. <http://doi.org/10.1016/j.energy.2006.01.007.>

Gu, B., Ge, Y., Ren, Y., et al., 2012. Atmospheric reactive nitrogen in China: sources, recent trends, and damage costs. Environ. Sci. Technol. 46, 9420–9427. http://doi.org/10.1021/es301446g.

IPCC. 2006. Guidelines for national greenhouse gas inventories. In: Eggleston, S., Buendia, L., Miwa, K., (Eds.), IPCC National Greenhouse Gas Inventories Programme. Institute for Global Environmental Strategies (IGES), Hayama, Japan, 664

Van Grinsven, H.J., Holland, M., Jacobsen, B.H., et al., 2013. Costs and benefits of nitrogen for Europe and implications for mitigation. Environ. Sci. Technol. 47, 3571–3579. http://doi.org/10.1021/es303804g.

Williams, A.G., Audsley, E., Sandars, D.L., 2006. Determining the environmental burdens and resource use in the production of agricultural and horticultural commodities. Final report to Defra on project IS0205. Retrieved from and www.agril ca.orgwww.defra.gov.uk

Xia, L.L., Ti, C.P., Li, B.L., et al., 2016. Greenhouse gas emissions and reactive nitrogen releases during the life-cycles of staple food production in China and their mitigation potential. Sci. Total. Environ. 556, 116–125. https://doi.org/10.1016/j.scitotenv.2016.02.204

Yuan, B.R., Nie, Z.R., Di, X.H., et al., 2006. Life cycle inventories of fossil fuels in China (Ⅰ): Energy sources consumption and direct pollutant emissions. Modern Chemical Industry. 26(3), 59–64. <https://doi.org/10.3321/j.issn:0253-4320.2006.03.015>. (In Chinese).

Yue, S.C., 2013. Optimum nitrogen management for high-yielding wheat and maize cropping system. China Agricultural University, Beijing, pp. 80 (In Chinese)

Zhang, W.F., Dou, Z.X., He, P.,et al., 2013. New technologies reduce greenhouse gas emissions from nitrogenous fertilizer in China. P. Natl. Acad. Sci. USA 110, 8375-8380. <http://doi.org/10.1073/pnas.1210447110>.
